# Infective Endocarditis Outcomes in Jeddah City, Saudi Arabia

**DOI:** 10.7759/cureus.20556

**Published:** 2021-12-20

**Authors:** Mohammed H Bogari, Aseel S Jarwan, Abdullah O Abukhodair, Basil A Alzahrani, Jawad A Alsayegh, Alaa Al-Kathiri, Abdulhalim J Kinsara

**Affiliations:** 1 Medicine and Surgery, College of Medicine, King Saud Bin Abdulaziz University for Health Sciences, Jeddah, SAU; 2 Laboratory Medicine/Molecular Medicine, College of Medicine, King Saud Bin Abdulaziz University for Health Sciences, Jeddah, SAU; 3 Cardiology, College of Medicine, King Saud Bin Abdulaziz University for Health Sciences, Ministry of National Guard Health Affairs, King Abdullah International Medical Research Center, Jeddah, SAU

**Keywords:** jeddah, staphylococcus aureus, microbiologic, duke criteria, culture positive, tertiary hospital, mitral valve, infective endocarditis

## Abstract

Objectives: This study aimed to analyze all the reported cases of definitive infective endocarditis, based on the modified Duke criteria in a tertiary hospital over the past five years, focusing on the causative organism/s, predisposing factors, and outcomes.

Methods: This is a cross-sectional retrospective study. Patients with a confirmed diagnosis of infective endocarditis using modified Duke criteria were included. The demographic data, predisposing factors, the causative microorganisms, laboratory and echocardiography results, and treatment were collected.

Results: In total, 37 patients were identified, 22 were male, and the median age was 59 years. Native valve endocarditis was found in 29 (78.3%) patients. The most frequently involved valves were the mitral valve in eight (42.1%) and aortic valve in six (31.6%) patients. Fever occurred in 22 patients (59.5%). The most frequent organisms were *Staphylococcus aureus* in 14 (37.4%) patients, coagulase-negative staphylococci in seven (18.9%) patients, and streptococci in seven (18.9%) patients. The majority (n=27) of the patients (72.97%) were treated medically, with 10 (27.02%) requiring in-hospital surgical intervention. The in-hospital mortality rate was 24.3%. Late presentation, reluctance to undergo surgery, and the past history of rheumatic fever were the contributing factors.

Conclusion: Native valve endocarditis is the major type of infective endocarditis. The most frequent organisms were *Staphylococcus aureus*, Streptococcus, and coagulase-negative staphylococci. In our study, infective endocarditis was more common among males, surgical intervention was low, and a high in-hospital mortality rate was noted in our series.

## Introduction

Infective endocarditis (IE) is a lethal microbial infection that targets native or prosthetic heart valves, the endocardial surface, and pacemaker or defibrillator leads, which may result in forming vegetation [1-3]. It infrequently settles in the chordae tendineae or interventricular septum [2]. Infective endocarditis is globally considered a rare disease, with an incidence of 3-10 per 100,000 population annually [4,5]. A study in Riyadh, Saudi Arabia, reported 502 cases in a period of 10 years (2009-2019) [6]. The most frequent organisms were *Staphylococcus aureus*, streptococci, and coagulase-negative staphylococci. Fungi and viruses were infrequent [1,7-9]. The mortality rate of patients with IE ranges between 10% and 30%, depending on the pathogen, the comorbidities of the patient, and the status of the valve, whether native or prostatic [10]. Diagnosing IE patients requires a good clinical schema. The modified Duke criteria, with high specificity and sensitivity, are the current gold standard for the diagnosis of IE [11-13]. This study aimed to address the causative organisms, predisposing factors, and outcome of all the reported cases with a definitive IE diagnosis based on the modified Duke criteria at a tertiary hospital in Jeddah, Saudi Arabia, over the past five years.

## Materials and methods

A cross-sectional retrospective study was conducted. This study was approved by the Institutional Review Board of King Abdullah International Medical Research Center (approval number: RJ19/083/J). The main objectives were to investigate the causative organisms that caused the IE and to analyze the patient outcomes in King Abdulaziz Medical City (KAMC-J), Jeddah, Saudi Arabia, from 2016 to 2021. KAMC has a 751-bed capacity. The patient data were collected from the electronic medical records. The modified Duke criteria were used to identify definitive IE patients. In total, 37 patients were included. The demographic data, predisposing factors, echocardiogram, and laboratory results, including the causative microorganisms, erythrocyte sedimentation rate (ESR), C-reactive protein (CRP) level, and the type of management were collected. The data were analyzed using the John Macintosh Project (JMP) software version 15 (Cary, NC: SAS Institute Inc.). The continuous variables are presented as mean and standard deviation and the categorical variables are as frequency and percentage.

The patient laboratory results are presented as median and interquartile range (IQR), instead of mean and standard deviation, due to the variation in numbers. The association between the outcomes and the type of organisms was analyzed with a chi-square test. A p-value less than 0.05 was considered significant.

## Results

In total, 37 patients met the inclusion criteria, 22 (59.5%) were male, the median age was 59 years, the mean heart rate was 99.6±32.2 beats per minute, and 15 patients had tachycardia. The mean temperature was 37.6±1.0ºC, and 22 (59.5%) patients had a high temperature >38ºC. The majority (78.3%, n=29) of sample had native valve involvement, and the rest had prosthetic IE. Of this group, four had both a prosthetic mitral and aortic valve involvement. The majority of IE cases were community-acquired, while five cases were suspected to be hospital-acquired. Just more than half (51.4%, n=19) of the sample had vegetation on the transthoracic echo. The most frequent site of vegetation was the mitral valve (42.1%, n=8), followed by the aortic valve (31.6%, n=6), and the tricuspid valve (21.1%, n=4). Only one patient had double valve involvement that was seen on the mitral and aortic valves. Almost half (48.6%) had no evidence of vegetation on the initial transthoracic echo but found on transesophageal echocardiography (TEE). TEE raised the sensitivity to 87%, making the test an important tool for early diagnosis and detecting complications. TEE also was helpful to detect the valve dehiscence in one out of eight cases with those who had prosthetic valves. Valves dysfunction was recorded on echocardiogram with new regurgitation in 32 (86.5%) and stenosis in six (16.2%) patients. The majority (72.9%, n=27) showed no calcification on the echocardiogram, but 10 (27.1%) patients had a calcified valve which was predominately on the mitral valve. Only one patient had a calcified pulmonary prosthetic valve (Table 1).

**Table 1:** Basic characteristics of patients HR: heart rate; MV: mitral valve; AV: aortic valve; TV: tricuspid valve; PV: prosthetic valve

Variables			Total number (N=37)
Age			51.7± 22.0
Height			152.1±32.9
Weight			66.3± 23.0
HR			99.6± 32.2
Temperature			37.6± 1.0
Gender	Male		22 (59.5)
	Female		15 (40.5)
Eco finding		Vegetation	19 (51.4)
		No vegetation	18 (48.6)
Area of vegetation		AV	6 (31.6)
		MV	8 (42.1)
		TV	4 (21.1)
		AV and MV	1 (5.3)
Type of valve		Native	29 (78.3)
		Prosthetic	8 (21.7)
Area of prosthetic valve		AV	3 (37.5)
		MV and AV	4 (50.0)
		PV	1 (12.5)
Valve stenosis		Yes	6 (16.2)
		No	31 (83.8)
Area of stenosis		AV	4 (66.7)
		MV	2 (33.3)
Valve regurgitation		Yes	32 (86.5)
		No	5 (13.5)
Calcification		AV	2 (5.4)
		MV	5 (13.5)
		MV and AV	1 (2.7)
		PV	1 (2.7)
		TV	1 (2.7)
		No	27 (72.9)

The majority of the organisms that caused IE were *Staphylococcus aureus* (n=14, 37.8%) (nine cases of methicillin-susceptible *Staphylococcus aureus* {MSSA} and five cases of methicillin-resistant *Staphylococcus aureus* {MRSA}) followed by Streptococcus (18.9%, n=7) (two cases of *Streptococcus gordonii*, three cases of *Streptococcus mitis*, one case of *Streptococcus anginosus*, and one case of *Streptococcus agalactiae* group B), coagulase-negative staphylococci (n=7, 18.9%), and *Enterococcus faecalis* (n=2, 5.4%). Seven (18.9%) IE patients had a positive blood culture for other microorganisms such as brucella, *Coxiella burnetii*, candida species (parapsilosis and glabrate), *Pseudomonas aeruginosa*, and Cutibacterium. A few laboratory tests were done for the samples, including C-reactive protein (CRP), erythrocyte sedimentation rates (ESR), and creatinine. The median level of CRP was 75 (IQR: 181.7), the ESR median level was 53.5 (IQR: 73.7), and the median creatinine level was 93 (IQR: 195) (Table 2).

**Table 2:** Patient laboratory results CRP: C-reactive protein; ESR: erythrocyte sedimentation rates; WBC: white blood cell; IQR: interquartile range

Parameter	Median	IQR
CRP	75.0	181.7
ESR	53.5	73.7
WBC	11.8	8.6
Platelets	215.0	240.7
Hemoglobin	10.2	2.9
Creatinine	93	195.0

Of the samples, 18 patients (48.5%) had a heart murmur, caused by *Staphylococcus aureus* (five patients) and Streptococcus (four patients). A portion (35.1%, n=13) had some signs of heart failure that was most frequently associated with *Staphylococcus aureus* organisms (four patients). A small proportion (18.9%, n=7) underwent surgery during hospitalization, with three deciding to postpone the surgery, 27 patients (72.97%) were treated only by medical therapy. Those who did not go to surgery were either advanced age, had a multi-organ failure, or did not accept the risk associated with surgery. However, surgical option is indicated only in a certain clinical scenario, like staph or multiple septic emboli or abscess formation. Regarding the mortality rate, nine patients (24.3%) died after the diagnosis of IE due to type of organism (*Staphylococcus aureus*) and multiorgan failure (Table 3).

**Table 3:** Outcomes by type of organism CoNS: coagulase-negative staphylococci

Outcome		N=37	Staphylococcus aureus	CoNS	Streptococcus	Enterococcus	Other	p-Value
Heart murmur	Yes	18(48.5)	5	3	4	2	4	0.47
	No	19(51.5)	9	4	3	0	3	
Heart failure	Yes	13(35.1)	4	3	0	2	4	0.051
	No	24(64.9)	10	4	7	0	3	
Intervention	Only medication	27(72.97)	12	5	5	2	3	0.72
	Antibiotic sensitivity	-	Floxacillin and cefazolin	Vancomycin	Ampicillin	Vancomycin and gentamicin	-	
	Surgery	10(27.03)	2	2	2	0	4	
Death	Yes	9 (24.3)	4	2	1	0	2	0.86
	No	28 (75.7)	10	5	6	2	5	

## Discussion

In this study, in five years, 37 cases met our inclusion criteria, with a mean age of 51.7 years and a male to female ratio of 1.5:1. A previous study in Jeddah included 60 patients over a period of 17 years; their mean age was 48.71 years and the male to female ratio was 1.2:1 [2]. In total, 37 patients had a positive blood culture, and the most frequent causative organisms in our study were *Staphylococcus aureus*, Streptococcus, and coagulase-negative staphylococci. These findings were similar to a local study done in Riyadh [14]. In our study, there was a rare organism, but reported one,* Coxiella burnetii*, which causes chronic endocarditis, secondary to Q fever [15]. Despite *Coxiella burnetii*, our results were similar in another study reporting the most prevalent organisms such as *Staphylococcus aureus*, 15 (25%) cases, and viridans streptococci, eight (13.3%) cases [2]. In the United States, the most prevalent causative organisms were the group of viridians streptococci, followed by *Staphylococcus aureus* [16]. The most affected valves were mitral and aortic valves. The main presenting symptom was fever (22/37), which is similar to a study done in Dunedin Public Hospital [17]. An increase in body temperature is an important feature that can be seen in patients with IE; however, the absence of fever does not exclude the possibility of IE. In a study done in the Aseer region, 22 patients (50%) required surgery [18]. The mortality rate in our study was 24.3%, while it was 29.4% in the study done in 2009 in Dhahran, and the mortality rate was 36.7% in the study conducted in Jeddah [19,2,20]. In fact, a systematic review revealed that one out of four cases of IE died inside the hospital [20]. The high mortality was linked to the type of organism, late presentation, and the development of multiorgan failure. The high mortality noted globally should address the importance of focusing on prevention, especially in valvular heart disease cases [21,22].

The mortality rate was higher in patients who had a positive blood culture of *Staphylococcus aureus* compared to the other microorganisms in our study (Table 3). The high mortality rate associated with *Staphylococcus aureus* may be due to a higher incidence of heart failure [23]. CRP and ESR play an important role in the prognosis of IE patients; a high level of these two markers is associated with high mortality and major complications. A review of literature of all studies conducted in the regions (Jeddah, Abha, Dhahran, and Riyadh) showed a similar pattern in terms of the most common organism, nature of the valve affected, and mortality rate (Table 4) [2,14,18,19]. The study was limited to being a single center but the accumulative data from other centers in the country will give the health authority a chance for taking the steps towards early diagnosis and improve the factors that adversely affect the outcomes. Our study should also alert the health authority to initiate a registry across the country as such registry will enable to improve the outcome of IE management. Such registry will define the barriers to improve the outcomes, such as the time to diagnosis, the nature of the infection (community vs. hospital), and the pattern of microbial resistance.

**Table 4:** Comparison with other local studies

Comparison	Current study	Alghamdi et al. [2]	Assiri [18]	Al-Tawfiq and Sufi [19]	Nashmi and Memish [14]
City	Jeddah	Jeddah	Abha	Dhahran	Riyadh
Year	2021	2021	2011	2009	2007
Sample size	37	60	44	54	47
Duration	2016-2021	2003-2019	2002-2007	1995-2008	1993-2003
Most frequent organism	Staphylococcus aureus	Staphylococcus aureus	Staphylococcus aureus	Staphylococcus aureus	Staphylococcus aureus
Most affected valve	Mitral	Mitral	Mitral	Mitral	Mitral
Mortality	24.30%	36.70%	No data	29.40%	8.50%

## Conclusions

Infective endocarditis is an uncommon disease. The majority of affected patients were male which is similar to many studies that confirm IE has a male predilection. The most offending organism is *Staphylococcus aureus*. One-quarter of affected patients underwent surgical intervention while being in hospital, before discharge. In our study, IE disease has a high mortality rate. However, a similar mortality rate was reported among different studies that were conducted in the region. We strongly believe that it is important to have a registry for all cases of IE across the country.
